# A survey of physician practices on the inpatient medical stabilization of patients with avoidant/restrictive food intake disorder

**DOI:** 10.1186/s40337-018-0212-4

**Published:** 2018-09-26

**Authors:** Carly E. Guss, Tracy K. Richmond, Sara Forman

**Affiliations:** Division of Adolescent/Young Adult Medicine, Boston Children’s Hospital, Department of Pediatrics, Harvard Medical School, 300 Longwood Avenue, Boston, MA 02115 USA

## Abstract

**Background:**

Avoidant/restrictive food intake disorder (ARFID) was added to the Diagnostic and Statistical Manual of Mental Disorders Fifth Edition in 2013. ARFID can result in impaired growth and significant nutritional deficiency; individuals with ARFID may be so nutritionally compromised that they require medical stabilization in a hospital. Prior to the new diagnostic criteria, it is unclear how patients now diagnosed with ARFID may have been medically stabilized when hospitalized. Our study aim was to assess the inpatient medical management of adolescents with ARFID.

**Methods:**

United States-based physician members of the Society for Adolescent Health and Medicine’s Eating Disorder Special Interest Group’s listserv or the National Eating Disorders Quality Improvement Collaborative were invited to participate in an anonymous survey regarding their practices of care for hospitalized patients with ARFID.

**Results:**

Thirty-seven (44.6%) of 83 physicians completed the survey; 73.0% (*n* = 27) of respondents medically admitted patients with ARFID. Half of respondents who admitted did not use any protocol for refeeding; 55% of those with a protocol used an anorexia nervosa treatment protocol. Solid food and nasogastric feeds were most commonly used for nutritional rehabilitation. Few typically prescribed medications in the hospital during medical stabilization.

**Conclusions:**

There is considerable variability of practice in the treatment of hospitalized patients with ARFID. An important next step is to test the efficacy of protocols for anorexia nervosa in treating ARFID patients.

## Plain ENGLISH summary

The diagnosis of Avoidant/Restrictive food intake disorder (ARFID) was added to the *Diagnostic and Statistical Manual of Mental Disorders Fifth Edition* in 2013. It has been used in order to describe patients with poor nutritional intake leading to weight loss and/or growth failure but without poor body image. These patients may need to be hospitalized, and there is currently very little research on how treatment of ARFID in the hospital may be different than patients with anorexia. This study surveyed physicians in the United States who care for patients with ARFID who require medical hospitalization. Respondents to our survey often used a multidisciplinary team to care for patients with ARFID and that nasogastric feeding tubes are often used. Moreover, the findings demonstrated that there is no standardized way to care for patients with ARFID, and many providers are relying on anorexia nervosa protocols. This variability in treatment demonstrates that further studies are needed to determine what protocols may be best used in this patient population.

## Background

Diagnosis and treatment of patients with eating disorders is evolving as evidenced by recent changes in eating disorder classifications in the *Diagnostic and Statistical Manual of Mental Disorders Fifth Edition* (DSM-5). Avoidant/restrictive food intake disorder (ARFID) is one of several new diagnoses added to the DSM-5 in 2013 [1]. ARFID emerged due to lack of an encompassing diagnosis to describe patients with inadequate nutritional intake but absent body dysmorphia [2]. ARFID is not a diagnosis limited to youth; ARFID may also be diagnosed in the adult population [3]. The publication of the DSM-5 and with it the definition of ARFID as a diagnosis has made the study of patients with ARFID easier as prior to the DSM-5 they may have been given several different diagnoses. Regardless of the etiology of ARFID, the diagnosis states that there are medical or psychosocial complications that require intervention [4]. While estimates of ARFID’s prevalence vary, one study of patients with eating disorders across several institutions found that 14% met the DSM-5 criteria for ARFID [5]. As ARFID can result in impaired growth and significant nutritional deficiency, these patients may require medical stabilization. Hospitalized patients with ARFID tend to be younger and require longer hospital stays than patients with other eating disorders leading to hospitalization [6, 7]. Prior to the new diagnostic criteria, it is unclear how patients now diagnosed with ARFID may have been medically stabilized when hospitalized. A recent retrospective chart review of patients assessed for an eating disorder found that more than half (57%) of patients diagnosed with ARFID had an inpatient hospitalization [8]. There are not published criteria for the admission of ARFID, which means that providers may rely on established AN criteria such as bradycardia, orthostatic hypotension, electrolyte abnormalities, and low weight [5, 6, 9, 10]. Descriptive studies cite reasons for hospitalization with a diagnosis of ARFID, although these are not necessarily criteria. For example, weight below 80% of goal, loss of over 20% of weight, failure of outpatient treatment, and bradycardia [8].

While methods for refeeding medically unstable patients with AN are described in clinical practice guidelines [9, 11, 12], there is a paucity of research published on inpatient medical treatment of patients with ARFID. The aim of this preliminary study was to determine the current protocols and practices used for inpatient medical stabilization of patients with ARFID in the United States.

## Methods

United States-based physician members of the Society for Adolescent Health and Medicine’s Eating Disorder Special Interest Group’s listserv or the National Eating Disorders Quality Improvement Collaborative [13] were invited to participate on three separate occasions via email with a link to the survey. If members were a part of both listservs, they received a single invitation. Respondents who did not admit patients with ARFID for nutritional rehabilitation in a hospital setting completed only the demographics portion of the survey. Participants were able to skip questions, so results have varying response rates. The anonymous online survey was based on our prior work assessing variability in inpatient management of patients with AN [14] and was exempt by the Boston Children’s Hospital Institutional Review Board due to the fact that it was an anonymous survey of clinicians. Study data were collected and managed by REDCap (Nashville, TN).

## Results

Thirty-seven of 83 eligible physicians completed the survey for a response rate of 44.6%. Based on response to a single survey question, all respondents reported that they were familiar with the diagnosis of ARFID. Of those who responded, 27 (73.0%) admitted patients with ARFID for nutritional rehabilitation. Providers who admitted patients with ARFID tended to be based in an academic medicine site and have specialization in Adolescent Medicine (Table 1).

**Table 1:** Demographic characteristics of physician respondents

Characteristic	All Respondents *n* = 37 *n* (%)	Respondents who Admit ARFID Patients for Medical Stabilization *n* = 27 *n* (%)
Sex		
Male	5 (13.5)	4 (14.8)
Female	25 (67.6)	16 (59.3)
No response	7 (18.9)	7 (25.9)
Residency Training		
Pediatrics	25 (67.6)	18 (66.7)
Medicine/Pediatrics	2 (5.4)	1(3.7)
No response	10 (27.0)	8 (29.6)
Adolescent Medicine Fellowship		
Yes	27 (73.0)	19 (70.3)
Years in practice as Attending Physician		
< 10	9 (24.3)	6 (22.2)
10–20	6 (16.2)	3 (11.1)
> 20	14 (37.8)	10 (37.0)
No response	8 (21.6)	8 (29.6)
Practice setting		
Academic	21 (56.8)	17 (63.0)
Group private practice	4 (10.8)	0 (0)
Health maintenance organization	2 (5.4)	0 (0)
Solo private practice	1 (2.7)	1 (3.7)
Community hospital	1 (2.7)	0 (0)
No response	8 (21.6)	8 (29.6)
Practice location		
Northeast	11 (29.7)	6 (22.2)
West	10 (27.0)	7 (25.9)
South	5 (13.6)	3 (11.1)
Midwest	3 (8.1)	3 (11.1)
No response	8 (23.6)	8 (29.6)

The majority of providers admitted patients with ARFID to a mixed pediatric and adolescent medical unit (Table 2). Most respondents who cared for patients with ARFID in an inpatient setting reported that their teams included mental health providers and a medical provider. The most common adjunctive therapies available during a medical admission were group therapy and nutritional education.

**Table 2:** Inpatient refeeding protocol and inpatient care team^a^

Characteristic	*n* (%)
Admission location (select all that apply)	*n* = 26
Mixed pediatric and adolescent medical unit	14 (51.9)
Adolescent medical unit	4 (14.8)
Adolescent psychiatric unit	3 (11.1)
Medical/psychiatric unit	3 (11.1)
Pediatric intensive care unit	1 (3.7)
Other	2 (7.4)
Specific refeeding protocol	*n* = 22
No	11 (50.0)
Yes	11 (50.0)
If yes, protocol same as anorexia protocol	6/11 (54.0)
Form of nutrition for refeeding (select all that apply)	*n* = 22
Food	20 (90.9)
Nasogastric feeds	11 (50.0)
Comfort foods	10 (45.4)
Liquid nutrition	10 (45.4)
Intravenous fluids	2 (9.1)
Total parenteral nutrition	1 (4.5)
Meals observed	*n* = 21
Yes	18 (85.7)
No	3 (14.3)
Patient weighed daily	*n* = 22
No	1 (4.5)
Yes	21 (95.5)
When phosphorus supplements prescribed	*n* = 22
Only if phosphorus drops	13 (59.1)
On all patients	7 (31.8)
Never	1 (4.5)
Other	1 (4.5)
Inpatient care team members (select all that apply)	*n* = 22
Adolescent medicine	21 (95.5)
Psychiatrist	19 (86.4)
Psychologist	16 (72.7)
Social work	13 (59.0)
Family based treatment therapist	5 (22.7)
Occupational therapist	4 (18.2)
General pediatrics	4 (18.2)
Family therapist	3 (13.6)
Feeding specialist	2 (9.1)
Medications are typically prescribed	*n* = 20
No	13 (59.1)
Yes	7 (35.0)
Which medications are prescribed (select all that apply)^b^	*n* = 6
Selective serotonin reuptake inhibitor	6 (100.0)
Multivitamin	4 (66.7)
Atypical antipsychotic	4 (66.7)
Benzodiazepines	4 (66.7)
Cyproheptadine	3 (50.0)
Fish oil	2 (33.3)

^a^Some n’s may not add up to 27 as respondents were may have skipped questions or were allowed to select more than one characteristic

^b^Respondents answered that medications are typically prescribed and then received additional questions about each medication. Answers in table reflect options of “yes, sometimes” and “yes, always”

With regards to providing nutritional rehabilitation to patients, only half of respondents (*n* = 11/22) reported having a standardized protocol (Table 2). Of those who did have a standard protocol, more than half of them (*n* = 6/11) used the same protocol for patients with ARFID and AN. Therefore, only 22.7% of respondents (*n* = 5/22) report having a non-AN refeeding protocol used for their patients with ARFID. “Regular food” was the most common form of nutrition used for nutritional rehabilitation among all providers. Feeding via nasogastric tubes was also commonly reported (50%) and was provided as the initial feeding regimen, a nocturnal supplement, or in some other manner. Only 25.9% (*n* = 7/27) providers indicated that medications were typically prescribed during admission. Of those that did prescribe, all respondents used selective serotonin reuptake inhibitors and two-thirds additionally prescribed atypical anti-psychotics.

Seven respondents replied to the question, “Is there anything else you want to share about your management of ARFID patients or your refeeding protocol (if you have one)?” The main themes from free text responses were that ARFID needs to be treated differently than AN and that use of a multidisciplinary approach with additional services such as behavior modification/exposure therapy is important. For example, on respondent said: “Patients with ARFID [are] generally only hospitalized if <75% MBW or medically unstable. Treatment is focused on exposure, [occupational therapy] more involved than with other ED patients, often will have a behavior modification program put in place by OT and psych.” Another respondent said, “patients with ARFID are treated on our specialized medical ED unit with strong multidisciplinary presence. The framework is our structured [Family Based Treatment]-based ED program but the treatment is very much individualized for these patients depending on their symptoms and presentation.” Another respondent noted that they “are struggling with trying to figure out how to provide services for [both patients with ARFID and AN] in our partial hospitalization program… we treat [patients with ARFID] like other AN patients who are admitted but will use the foods they are comfortable with rather than feeding them everything like the AN patients.”

## Discussion

This preliminary study is the first of its kind to examine inpatient medical provider management practices for medical stabilization of patients with ARFID. We found that providers were rarely using standardized refeeding protocols aimed at ARFID. Although patients with ARFID differ significantly from patients with AN [1], half of medical providers using protocols relied on AN protocols. Providers’ concerns in open text responses reflect the need for research to clarify the efficacy of various treatments. This reflects how new ARFID is as diagnosis and subsequent lack of evidence for effective treatment. Moreover, few providers were prescribing medications to treat patients. There currently are not evidence-based pharmacologic treatments for ARFID [15]. In this survey providers who prescribed medications more commonly used SSRIs, although a recent case report notes that an adolescent patient was successfully treated for ARFID with buspirone [16], demonstrating that more research to optimize pharmacological interventions is needed. A commonality among providers was the incorporation of a multidisciplinary team, which was previously found to be of benefit [17]. Additionally, nutritional rehabilitation was frequently achieved with food and nasogastric tubes, although the efficacy of nasogastric tubes in this population is unknown. One retrospective review of outcomes of an inpatient protocol for medical stabilization of eating disorders found that patients with ARFID were significantly more likely to require nasogastric tube feeding compared to other eating disorder diagnoses [18]. These results stress the need for assessment and evaluation of the efficacy of currently used treatment modalities for patients with ARFID.

The strengths of this preliminary study include the variability in practice experience and geographic location of the respondents and the wide range of years in practice of the physicians who participated in the survey. Additionally, this was an investigation of current practices from providers who actively treat ARFID patients. There are limitations to our study. We had an overall response rate of 44.6%, and of the respondents not all admitted patients with ARFID for inpatient nutritional rehabilitation. Thus, the resulting sample was small and limits further analysis of results. The sample of providers is adolescent medicine-focused so results may not reflect the management of younger children with ARFID. We believe if there is bias in our results, they would overstate the use of protocols and common practices given the source for our sample. Respondents were not asked if patients were admitted to other services, such as gastroenterology, where other specialties may treat these patients differently. However, one retrospective study of gastroenterology clinics found only 1.5% of referral patients had ARFID, so it is perhaps less likely they would be admitted to gastroenterology [19]. Additionally, more specific measures of medical treatment may not be captured by this survey and there is no further information regarding the intricacies of the protocols developed by providers. Further research should seek to describe specific information for discharge criteria for ARFID patients as well as recommended post-discharge treatment after an inpatient stay for development of future protocols. A comparison of low-weight patients with ARFID to patients with AN demonstrated similar improvements in a partial hospitalization program, suggesting that there are areas of treatment that may overlap between the two diagnoses [20]. However, it is important to have a baseline for how ARFID patients are treated in inpatient settings before developing new protocols.

## Conclusion

In conclusion, the variability in the treatment approaches to the inpatient medical stabilization of patients with ARFID highlights the evidence gap regarding optimal treatment of this disorder. This may help explain why ARFID may result in longer hospital stay [21]. Additionally, as of now, there is a lack of randomized control trials to establish evidence-based treatment of ARFID, including studies to support the use of medication in either an inpatient or outpatient setting [4]. It may be that no single treatment works best for all ARFID patients, although as evidenced by this survey a multidisciplinary team is likely important to the effectiveness of treatment [21]. Development of future protocols should consider the socio-culture background and values of patients [22] as well as the differences between typical patients with ARFID and AN (such as younger age, prevalence of history of choking/gagging episode, and lack of body dysmorphia in those with ARFID in contrast to those with AN). An important next step is to assess the effectiveness of current protocols used in the treatment of patients with ARFID across programs.

## Abbreviations

AN: Anorexia nervosa
ARFID: Avoidant/restrictive food intake disorder
DSM-5: *Diagnostic and Statistical Manual of Mental Disorders Fifth Edition*
